# Quantum retrodiction

**DOI:** 10.1098/rsta.2023.0338

**Published:** 2024-12-24

**Authors:** John Jeffers, Daniel K. L. Oi, Thomas Brougham

**Affiliations:** ^1^ Department of Physics, University of Strathclyde, John Anderson Building, 107 Rottenrow, Glasgow G4 0NG, UK

**Keywords:** retrodiction, measurement, detection

## Abstract

Quantum retrodiction, in which the state of a quantum system prior to a measurement is assigned based on the results of that measurement, has had a long history and has been used in quantum optics research for decades. Here we summarize the theory and point out some of the more interesting results, before applying the theory to state identification from multiple shots of an experiment. One surprising result is that we show that a photodetector with low quantum efficiency can discriminate between photonic states better than a detector with a higher efficiency.

This article is part of the theme issue ‘The quantum theory of light’.

## 1. Introduction

Our world is ordered in time. Not only is it ordered, but we also perceive an apparent arrow. We often have information about a consecutive set of events that we call the past, but we have no such information about other consecutive events, which we call the future. We have to predict the future using a set of physical laws.

However, the laws of physics for closed systems show no such time-directionality. This does not matter for many classical physical systems. Given a set of conditions at a particular time we can solve the equations of motion either forwards in time to get the future state of the system, or backwards in time to find out about the past, if we do not know it. We build directionality into these solutions via the principle of (strong) causality, which states that effect follows cause, or more generally that no event can have an effect on anything outside its future light cone. A weaker causality principle is often applied to state preparation and measurement in quantum physics, one which states simply that information cannot travel outside the light cone.

The appeal of the strong causality principle can bias our theories. This bias has, in the past, led to a sense of the immutability of probability that was partly behind the slow path to the acceptance of Bayesianism in statistics [1–3]. It is certainly inherent in the standard formalism of quantum physics, in which a prepared state evolves forwards in time and is measured later by a measuring device. The bias of strong causality provides the evolved prepared state with a sheen of apparent realism, but affects nothing measurable. We call the standard formalism predictive quantum mechanics to distinguish it from the retrodictive formalism that we will introduce and describe. Retrodiction in quantum physics has a long history [4–6], reignited in the late 1990s as quantum optics experimental techniques began to catch up with earlier theoretical progress. Projection synthesis experiments became possible, which led to the development of the quantum scissors device [7], the internal measurements of which can be interpreted as sending a quantum state backwards in time to form the output of the device. Applied more widely, this principle led to the basics of retrodictive quantum theory [8–10]. Formalism has since been applied in both theory and experiments to retrodicting atomic states [11,12], quantum optical communication [13–15], micromaser field measurements [16,17], quantum state engineering [18,19], fidelities in postselection [20], generalized measurements [21], quantum imaging experiments [22–26], the mean king problem [27], a generalization of the Gleason–Busch theorem [28], photon trajectories in interferometers [29], interspersed with developments of the fundamental theory [30,31] and the discovery of the retrodictive master equation [32]. The more philosophical aspects of the theory were covered in [33]. More recently there have been significant applications to quantum information, providing a retrodictive fluctuation theorem and entropy bounds [34,35], to conditional expectations and smoothing [36], in quantum computing [37], to time-reversal symmetry with priors [38] and to the photostatistics of Gaussian states [39].

This paper is organized as follows. In §2, we briefly describe the standard predictive formalism of quantum physics and then move to the retrodictive form of the theory. In the next section, we show the results of applying the theory to optical attenuation and amplification. We will necessarily be brief, concentrating on results rather than detailing the full calculations, but will direct the reader to the appropriate references for these. In §4, we consider applying the principles of retrodictive quantum physics to multiple shots of an experiment, deriving new results that provide an intuitive picture of how we find information about a quantum state, before presenting our conclusions.

## 2. Predictive and retrodictive quantum mechanics

Consider a system that is prepared at some time $t_{p}$ in a quantum state |ψ⟩ and later (at $t_{m}$) measured to be in a quantum state |ϕ⟩. If no evolution occurs, then in predictive quantum mechanics the state of the system between the preparation and measurement times is |ψ⟩. At $t_{m}$, the Copenhagen-inspired collapse occurs and the state changes abruptly. Quantum physics allows us to calculate the probability that the measured state is |ϕ⟩ as


(2.1) $$\begin{array}{l} |\langle \phi |\psi \rangle {|}^{2}. \end{array}$$

**Figure 1.**
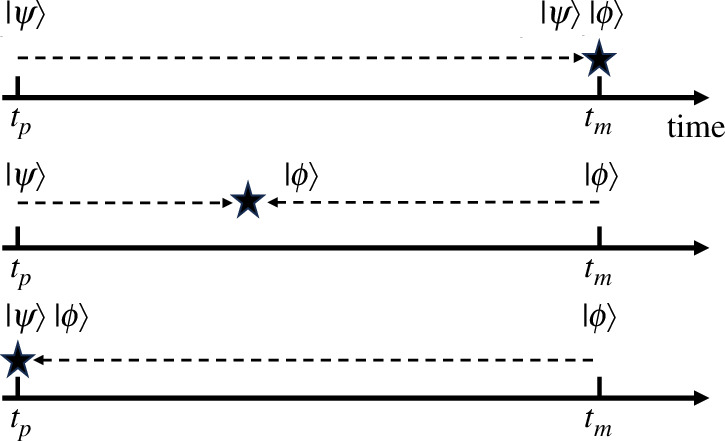
Schematic showing the standard predictive viewpoint with a collapse at the measurement time (top), an intermediate viewpoint (middle) and the fully retrodictive one (bottom), where the collapse occurs immediately after preparation.

So far—so standard, but we note now that there is no access allowed to the state between the two times. Any extraction of information about the state between the preparation and measurement events would amount to an intermediate measurement, so in reality we know nothing about the state between the two events. If such a collapse occurred, it could have done so at any time, without affecting the probability. This collapse-time independence of probability is what allows us to choose, if we wish, the *retrodictive* formalism, in which the collapse occurs at the preparation time. The state between preparation and measurement is then the measured state |ϕ⟩ (figure 1).

### (a) Probabilities in predictive quantum mechanics

More generally we can describe a thought experiment in which a preparation device operated by Alice prepares quantum states of a system that are later measured by a measurement device owned by Bob. We follow here a set of arguments that first appeared in [10]. We assume that the system evolves between preparation and measurement, but neither closed nor open system evolution changes the structure of the theory, so we leave its explicit consideration for now. The set of possible states prepared by the device is $\left\{{\hat{\rho }}_{i}^{\text{ pred }}\right\}$. In a single shot of the experiment, one of the set is prepared with *a priori* probability $p_{i}^{\text{ ap }}$ and the preparation outcome, i, is noted by Alice (throughout this manuscript the subscript i always refers to a preparation outcome). The superscript reminds us that the state is the one that we would normally assign to the system in predictive quantum mechanics. Bob does not know the preparation outcome, but he measures the state of the system that he receives. Prior to his measurement his best guess of the prepared state is the *a priori* density operator,


(2.2) $$\begin{array}{l} {\hat{\rho }}_{\text{ ap }}=\sum_{i}p_{i}^{\text{ ap }}{\hat{\rho }}_{i}^{\text{ pred }}. \end{array}$$

We say that the preparation is unbiased if this density operator is proportional to the identity operator for the system, but this will not normally be the case.

Bob’s measurement device has outcomes j (similarly, throughout this manuscript the subscript j always refers to a measurement outcome) that correspond to one of a set of positive, hermitian probability operators^1^ $\left\{{\hat{\pi }}_{j}\right\}$. In a single shot of the experiment, the state is measured and the measurement outcome, j, is noted by Bob. We normally require there to be a measurement result, no matter the prepared state, so this renders Bob’s measurement unbiased, which provides a condition on the measurement probability operators,


(2.3) $$\begin{array}{l} \sum_{j}{\hat{\pi }}_{j}=\hat{I}. \end{array}$$

In predictive quantum mechanics, the central task is to determine the probabilities of later measurement results given the preparation outcome. We can do this via the predictive conditional probability that Bob obtains the measurement result j, given that Alice’s preparation event was i,


(2.4) $$\begin{array}{l} p(j|i)=Tr[{\hat{\rho }}_{i}^{\text{ pred }}{\hat{\pi }}_{j}], \end{array}$$

which is effectively the standard Born probability rule.

### (b) Probabilities in retrodictive quantum mechanics

In retrodictive quantum mechanics, the central task is to determine the probabilities of earlier preparation outcomes given the result of a measurement, in other words to determine retrodictive conditional probabilities. We would like to be able to write these in terms of a retrodictive state based on the measurement result. To clarify where the different symmetries of the system enter, we first consider the situation where the preparation device is unbiased. Then we can use the *a priori* density operator to define a preparation device version of the measurement device probability operator,


(2.5) $$\begin{array}{l} {\hat{\xi }}_{i}=Dp_{i}^{\text{ ap }}{\hat{\rho }}_{i}^{\text{ pred }}, \end{array}$$

with D the system state space dimension, such that


(2.6) $$\begin{array}{l} \sum_{i}{\hat{\xi }}_{i}=D\sum_{i}p_{i}^{\text{ ap }}{\hat{\rho }}_{i}^{\text{ pred }}=\hat{I}. \end{array}$$

We would like to use this operator to write a retrodictive conditional probability based on a retrodictive state in the same way as in equation (2.4),


(2.7) $$\begin{array}{l} p(i|j)=Tr\left({\hat{\rho }}_{j}^{\text{ retr }}\xi_{i}\right), \end{array}$$

so that the differing temporal orders implied by the ordering of the indices in equations (2.7) and (2.4) align with the idea that we predict future events based on past ones and retrodict in the opposite temporal order. Bayes’ theorem [1,3,40] states that the retrodictive and predictive conditional probabilities are related by


(2.8) $$\begin{array}{l} p(i|j)=\frac{p(j|i)p_{i}^{\text{ ap }}}{p_{j}^{\text{ meas }}}, \end{array}$$

where $p_{j}^{\text{ meas }}$ is the prior probability of measurement result j. We simply substitute equation (2.4) and use equations (2.5) and (2.6) noting that $p_{j}^{\text{ meas }}$ can be written


(2.9) $$\begin{array}{l} p_{j}^{\text{ meas }}=\frac{Tr{\hat{\pi }}_{j}}{D} \end{array},$$

to find that we can indeed write the retrodictive conditional probability in the form of equation (2.7), provided that we define the retrodictive state as


(2.10) $$\begin{array}{l} {\hat{\rho }}_{j}^{\text{ retr }}=\frac{{\hat{\pi }}_{j}}{Tr{\hat{\pi }}_{j}}, \end{array}$$

simply a renormalized form of the probability operator.

For biased sources, which do not satisfy equation (2.6) we can define a set of operators,


(2.11) $$\begin{array}{l} {\hat{\lambda }}_{i}=p_{i}^{\text{ ap }}{\hat{\rho }}_{i}^{\text{ pred }}, \end{array}$$

and again use Bayes’ theorem and our definition of the retrodictive state to write


(2.12) $$\begin{array}{l} p(i|j)=\frac{Tr\left({\hat{\rho }}_{j}^{\text{ retr }}{\hat{\lambda }}_{i}\right)}{\sum_{k}Tr\left({\hat{\rho }}_{j}^{\text{ retr }}{\hat{\lambda }}_{k}\right)}. \end{array}$$

Note the lack of symmetry with the predictive conditional probability in equation (2.4). This is not any form of intrinsic time asymmetry in quantum mechanics, but is one imposed by the boundary condition of the biased source. We could equally well use a biased measurement and obtain fully symmetric forms of both the predictive and retrodictive conditional probabilities [30,31], but the added complication is not required here.

### (c) Evolution

Evolution between preparation and measurement is easily accommodated. Both closed and open systems evolution in retrodiction are based on the principle of collapse-time independence.

#### (i) Closed systems

**Figure 2.**
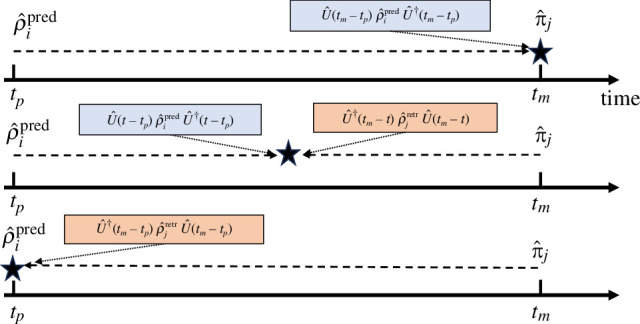
Schematic showing how evolution affects the states for a closed system in the predictive picture (top), an intermediate picture (middle) and the retrodictive picture (bottom).


Consider figure 2, which shows the standard predictive picture in which a state is prepared at time $t_{p}$ and evolves forwards in time until the collapse at the measurement time $t_{m}$. As the system is closed, the evolution is unitary. The predictive state evolved forwards in time to the measurement time is

(2.13) $$\begin{array}{l} {\hat{\rho }}_{i}^{\text{ pred }}(t_{m})=\hat{U}(t_{m}-t_{p}){\hat{\rho }}_{i}^{\text{ pred }}(t_{p}){\hat{U}}^{†}(t_{m}-t_{p}), \end{array}$$

where for a system with a time-independent Hamiltonian $\hat{H}$ the unitary evolution operator is


(2.14) $$\begin{array}{l} \hat{U}(t)=e^{-i\hat{H}t\;/\;\hbar } \end{array}.$$

The system evolves according to the Schrödinger equation for a duration $t_{m}-t_{p}$ before measurement. The predictive conditional probability is given by the evolved version of equation (2.4),


(2.15) $$\begin{array}{l} p(j|i)=Tr[{\hat{\rho }}_{i}^{\text{ pred }}(t_{m}){\hat{\pi }}_{j}]. \end{array}$$

Note that we can use the cyclic property of the trace to push the evolution on to the probability operator, where it becomes reverse time evolution and we can write the probability operator, reverse time-evolved to the preparation time as


(2.16) $$\begin{array}{lll} {\hat{\pi }}_{j}(t_{p}) & = & {\hat{U}}^{†}(t_{m}-t_{p}){\hat{\pi }}_{j}\hat{U}(t_{m}-t_{p}) \end{array}$$

(2.17) $$\begin{array}{lll} & = & Tr\left[{\hat{\pi }}_{j}\right]{\hat{\rho }}_{j}^{\text{ retr }}(t_{p}), \end{array}$$

so that we can write the retrodictive conditional probability as


(2.18) $$\begin{array}{l} p(i|j)=\frac{Tr\left({\hat{\rho }}_{j}^{\text{ retr }}(t_{p}){\hat{\lambda }}_{i}\right)}{\sum_{k}Tr\left({\hat{\rho }}_{j}^{\text{ retr }}(t_{p}){\hat{\lambda }}_{k}\right)} \end{array},$$

with a collapse occurring at the preparation time. A simpler formula, analogous to equation (2.7), can be written for unbiased sources. We further note that we could have chosen to divide the evolution at some intermediate collapse time so that


(2.19) $$\begin{array}{l} \hat{U}(t_{m}-t_{p})=\hat{U}(t_{m}-t)\hat{U}(t-t_{p}). \end{array}$$

Then part of the evolution can be backwards from the measurement time and another part forwards from the preparation, each to the collapse time t in the middle. All probabilities remain unaffected no matter what this time is. We can even include the evolution as part of either the preparation or the measurement devices.

#### (ii) Open systems

Open system evolution is not unitary. It causes pure initial states to lose information to the environment to become mixed. This renders it not time reversible, which would appear to make it more challenging for retrodiction. The evolution of density operators forwards in time is normally described using a Liouville–von Neumann master equation for the density operator which, for Born-Markov type evolution takes the form


(2.20) $$\begin{array}{l} {\dot{\hat{\rho }}}_{i}^{\text{ pred }}=\frac{-i}{\hbar }\left[\hat{H},{\hat{\rho }}_{i}^{\text{ pred }}\right]+\sum_{q}\left[2{\hat{A}}_{q}{\hat{\rho }}_{i}^{\text{ pred }}{\hat{A}}_{q}^{†}-{\hat{A}}_{q}^{†}{\hat{A}}_{q}{\hat{\rho }}_{i}^{\text{ pred }}-{\hat{\rho }}_{i}^{\text{ pred }}{\hat{A}}_{q}^{†}{\hat{A}}_{q}\right] \end{array},$$

where $\hat{H}$ is the system Hamiltonian and ${\hat{A}}_{q}$ is a system operator. We can now use the principle of collapse-time independence to derive a retrodictive version of the master equation. We follow the argument given in [32]; the reader should consult there for more details. We begin by noting that the predictive conditional probability given by equation (2.4) should not depend on the collapse time. This means that


(2.21) $$\begin{array}{l} \frac{\partial }{\partial t_{c}}p(j|i)=\frac{\partial }{\partial t_{c}}Tr[{\hat{\rho }}_{i}^{\text{ pred }}(t_{c}){\hat{\pi }}_{j}(t_{c})]=0, \end{array}$$

where the predictive density operator is evolved forwards in time from the preparation time and the probability operator is evolved backwards in time from the measurement time, each to the collapse time t, somewhere in the middle. This means that


(2.22) $$\begin{array}{l} Tr[\frac{\partial }{\partial t}\left({\hat{\rho }}_{i}^{\text{ pred }}(t)\right){\hat{\pi }}_{j}(t)]=-Tr[{\hat{\rho }}_{i}^{\text{ pred }}(t)\frac{\partial }{\partial t}\left({\hat{\pi }}_{j}(t)\right)]. \end{array}$$

We can use the predictive master equation (2.20) for the derivative on the left-hand side and then employ the cyclic property of the trace to see that the evolution equation for the probability operator is


(2.23) $$\begin{array}{r} \frac{\partial }{\partial t}{\hat{\pi }}_{j}=-\frac{i}{\hbar }\left[\hat{H},{\hat{\pi }}_{j}\right]+\sum_{q}\left[2{\hat{A}}_{q}^{†}{\hat{\pi }}_{j}{\hat{A}}_{q}-{\hat{A}}_{q}^{†}{\hat{A}}_{q}{\hat{\pi }}_{j}-{\hat{\pi }}_{j}{\hat{A}}_{q}^{†}{\hat{A}}_{q}\right]. \end{array}$$

This form is such that it ensures that at all times the sum of all probability operators is the identity, according to equation (2.3). To obtain the retrodictive master equation, we normalize according to equation (2.10), to obtain


(2.24) $$\begin{array}{lll} \frac{\partial }{\partial t}{\hat{\rho }}_{j}^{\text{ retr }} & = & -\frac{i}{\hbar }\left[\hat{H},{\hat{\rho }}_{j}^{\text{ retr }}\right]+\sum_{q}\left[2{\hat{A}}_{q}^{†}{\hat{\rho }}_{j}^{\text{ retr }}{\hat{A}}_{q}-{\hat{A}}_{q}^{†}{\hat{A}}_{q}{\hat{\rho }}_{j}^{\text{ retr }}-{\hat{\rho }}_{j}^{\text{ retr }}{\hat{A}}_{q}^{†}{\hat{A}}_{q}\right]\\ & - & 2{\hat{\rho }}_{j}^{\text{ retr }}Tr\left\{{\hat{\rho }}_{j}^{\text{ retr }}\sum_{q}[{\hat{A}}_{q}^{†},{\hat{A}}_{q}]\right\}, \end{array}$$

where the nonlinear term in the retrodictive density operator is a consequence of the renormalization. This master equation preserves the trace of the retrodictive density operator and ensures that it is non-negative definite. We solve it in the reverse time direction. Finally in this section, we note that there are other approaches that treat open systems retrodictive evolution more in the manner of closed systems based on thermofields [41] and on Fano diagonalization [42], but the master-equation approach described above is in more common usage.

## 3. A simple example: optical amplification and attenuation

We show in this section one example of the application of retrodiction to a simple quantum optical system. There are many others in the references. This particular example will provide some of the background for the next section on multiple measurements.

In classical physics noiseless optical amplification and attenuation are reverse processes. They each result in multiplying the optical signal by a factor—the gain G or the loss K. One can be used to undo the effect of the other on an optical field if the gain and loss of the separate two devices are related by G=1/K. This is not the case in quantum physics. Some very general considerations mean that deterministic amplification cannot be performed without adding extra noise photons to the signal, a minimum average number of G−1 [43–46]. This automatically degrades signal recovery if we try to reamplify an attenuated signal but, in fact, it simply guarantees that an amplifier cannot recreate (or clone) information lost to the environment. The amplifier and attenuator models that we describe were first introduced by Glauber [47] and are described in more detail elsewhere [48,49]. Here we consider the retrodictive forms of these processes and find a surprising, somewhat pleasing, equivalence that restores the reverse natures of amplification and attenuation in quantum physics. We then apply this to the detection of optical signals by an imperfect detector.

We begin by noting that the predictive master equations for an optical attenuator and amplifier are


(3.1) $$\dot{\hat{\rho }}(t)=\gamma N(T)\left(2{\hat{a}}^{†}\hat{\rho }\hat{a}-\hat{a}{\hat{a}}^{†}\hat{\rho }-\hat{\rho }\hat{a}{\hat{a}}^{†}\right)+\gamma \left[N(T)+1\right]\left(2\hat{a}\hat{\rho }{\hat{a}}^{†}-{\hat{a}}^{†}\hat{a}\hat{\rho }-\hat{\rho }{\hat{a}}^{†}\hat{a}\right),$$

for the attenuator and


(3.2) $$\dot{\hat{\rho }}(t)=\gamma \left[N(T)+1\right]\left(2{\hat{a}}^{†}\hat{\rho }\hat{a}-\hat{a}{\hat{a}}^{†}\hat{\rho }-\hat{\rho }\hat{a}{\hat{a}}^{†}\right)+\gamma N(T)\left(2\hat{a}\hat{\rho }{\hat{a}}^{†}-{\hat{a}}^{†}\hat{a}\hat{\rho }-\hat{\rho }{\hat{a}}^{†}\hat{a}\right),$$

for the amplifier, where in these equations we have suppressed the superscript denoting that they apply to predictive density operators only. The parameter γ is related to the attenuator loss, $K=e^{-2\gamma t}$ and the amplifier gain, $G=e^{2\gamma t}$. The factor N(T) is a thermal excitation function


(3.3) $$N(T)=\frac{1}{e^{\hbar \omega \;/\;k_{B}T}-1}\geq 0,$$

which provides the excess noise in the attenuator and amplifier, characterized by the optical angular frequency and a noise temperature T. Both γ and N(T) encode the physical attributes of the amplifier and are particular to each device. An amplifier (attenuator) with a larger γ amplifies (attenuates) more quickly and one with a larger N adds more noise. The equations above seem quite symmetrical, but their effect is not. If a vacuum state is attenuated we can solve equation (3.1) to see that the output of the device is noise photons, a thermal state of mean photon number N(1−K),


(3.4) $$\rho_{\text{ att }}^{\text{ pred }}=\frac{1}{N(1-K)+1}\sum_{n=0}^{\infty }{\left(\frac{N(1-K)}{N(1-K)+1}\right)}^{n}|n\rangle \langle n|,$$

which can be simply the vacuum state if no excess noise photons are added (N=0). Conversely if a vacuum state is amplified, the solution to equation (3.2) shows that the output is also a thermal state, but with a mean photon number (N+1)(G−1), which has non-zero minimum G−1 for N=0.

A simple application of the procedure leading to equation (2.24) to the equation for the attenuator gives


(3.5) $$\begin{array}{lll} {\dot{\hat{\rho }}}^{\text{ retr }}(t) & = & \gamma \left[N(T)+1\right]\left(2{\hat{a}}^{†}{\hat{\rho }}^{\text{ retr }}\hat{a}-\hat{a}{\hat{a}}^{†}{\hat{\rho }}^{\text{ retr }}-{\hat{\rho }}^{retr}\hat{a}{\hat{a}}^{†}\right)\\ & + & \gamma N(T)\left(2\hat{a}{\hat{\rho }}^{\text{ retr }}{\hat{a}}^{†}-{\hat{a}}^{†}\hat{a}{\hat{\rho }}^{\text{ retr }}-{\hat{\rho }}^{\text{ retr }}{\hat{a}}^{†}\hat{a}\right), \end{array}$$

which has precisely the same format as equation (3.2). If we had started with this predictive equation we would have found a retrodictive form identical to equation (3.1). This shows our equivalence; the detailed proof is found in the appendix of [14]. The above forms of predictive and retrodictive master equation are required to guarantee that the conditional probabilities are consistent. There are other simple proofs of the equivalence based on comparison of matrix elements [13,14].

**Figure 3.**
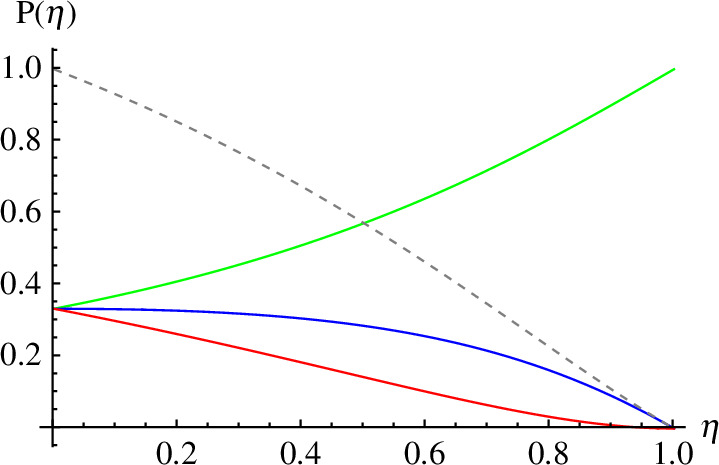
Input photon number state probability when the detector does not fire as a function of detector quantum efficiency for equal prior probabilities of input states. Solid curves are, from top to bottom (green, blue, red), the probabilities that 0, 1 or 2 photons were prepared. At the high-loss end all three states are equally likely. The dashed curve is the best estimate of the mean photon number of the input.

The equation for the retrodictive attenuator is particularly useful, as it can be applied directly to detection by an inefficent photodetector. If we detect a particular number of photocounts at a detector of quantum efficiency η, then to find the retrodictive state just prior to the detector we simply amplify the state (adding the requisite amplifier noise). So if an inefficient detector that adds no dark counts does not fire, this amounts to detecting in the thermal state


(3.6) $$\begin{array}{l} {\hat{\rho }}_{\text{ det }}^{\text{ retr }}=\frac{1}{G}\sum_{n=0}^{\infty }{\left(\frac{G-1}{G}\right)}^{n}|n\rangle \langle n|=\eta \sum_{n=0}^{\infty }(1-\eta {)}^{n}|n\rangle \langle n|, \end{array}$$

where η=1/G [8]. Any number of photons could have led to the detector not firing. If we now add in preparation information we can find the retrodictive conditional probability that particular states were prepared. Suppose, for example that only 0, 1 or 2 photons could have been prepared, each with equal prior probability 1/3. We plot in figure 3 the retrodictive conditional probability that each of these states was prepared, given that a detector of quantum efficiency η did not fire. We see that for a perfect detector the vacuum state was prepared with certainty. In the limit that the detector is very poor it becomes more likely that the one- and two-photon states were prepared and that they converge on to the prior probabilities in the poor-detector limit. Also plotted is the estimate of the mean photon number of the input. This is zero for a perfect detector and grows as η decreases. It would be infinite for η=0 (corresponding to the infinitely amplified vacuum state) but is suppressed by the limited state space.

## 4. Retrodiction of prepared states from multiple measurements

As stated in §2b, the basic task of retrodictive quantum mechanics is to use the measured state to determine the probability that a particular state was prepared. It is normally applicable to single shots of an experiment, for which a retrodictive state can be assigned. If multiple shots of an identical experiment are performed then different measurement outcomes can occur in different shots and so different retrodictive states would be assigned each time. As we shall see, however, the basic task can still be accomplished. There are two possible situations to consider. In the first scenario, the probabilistic choice of prepared state is made once, at the beginning. Afterwards the same preparation outcome occurs each time. This can occur by deliberate design, but it is also the normal situation for channel discrimination in a quantum communication setting. The alternative situation has multiple independent shots of the experiment with the same *a priori* density operator, but producing a new independent state consistent with this density operator each time. We consider the first of these situations, before commenting briefly on the second at the end of the section.

Suppose that we perform two experiments in which *the same state* was produced by the preparation device but with, in principle, different measurement outcomes 1 and 2. We must treat each measurement as an equally good provider of information about the prepared state. Therefore the best guess of the prepared state after the two shots’ shot is simply an equal mixture of the two measured retrodictive states


(4.1) $${\hat{\rho }}^{\left(2\right)}=\frac{1}{2}\left({\hat{\rho }}_{1}^{\text{ retr }}+{\hat{\rho }}_{2}^{\text{ retr }}\right)=\frac{1}{2}\left(\frac{{\hat{\pi }}_{1}}{Tr{\hat{\pi }}_{1}}+\frac{{\hat{\pi }}_{2}}{Tr{\hat{\pi }}_{2}}\right),$$

where the superscript on the state indicates that this is the best guess of the state of each shot after two measurements. The extension to more shots of the same identical state experiment is straightforward. If there are N shots and outcome j occurs $N_{j}$ times, the state is


(4.2) $${\hat{\rho }}^{\left(N\right)}=\frac{1}{N}\sum_{j}N_{j}{\hat{\rho }}_{j}^{\text{ retr }},$$

where $N=\sum_{j}N_{j}$. The quantity $N_{j}\;/\;N$ is our best, relative frequency-based, guess of the probability that in further shots of the experiment we would obtain the result j. It is not necessarily the same as the probability that the source produced state ${\hat{\rho }}_{j}^{\text{ retr }}$, because we have not used knowledge of which states could have been prepared. The state ${\hat{\rho }}^{\left(N\right)}$ is the best guess of the state produced in each of the N individual shots of the experiment. It is a necessarily limited guess because it does not include any prior information about the source, neither the possible prepared states nor their associated prior probabilities. In the following sections, we show how we can obtain a better guess of the prepared state using Bayesian reasoning after each shot of the experiment.

### (a) Prior information and conditional probabilities

The retrodictive states above are mixtures of the states that are measured. The situation changes if we have prior information about which states could have been prepared, even though the retrodictive state in each shot of the experiment does not. We know the (biased or unbiased) set of states that could have been prepared. If we have a source that produces a set of states ${\hat{\rho }}_{i}^{\text{ pred }}$ with prior probabilities $p_{i}^{\text{ ap }}$, the *a priori* density operator is given by equation (2.2). This is the best guess of the state produced each time by the preparation device with no knowledge of anything other than the states that could have been prepared and their prior probabilities. Can we use our measurement to adjust our best guess of the state prepared when we have no knowledge of the preparation outcome? The retrodictive states above will provide a guide to the possible effects on our knowledge of the prepared state.

### (b) Updating the prior state

We wish, by measurement, to retrodict the preparation event and hence the state that was prepared by the source, based on both our prior knowledge of the source and on our measurement result. We do not know the particular outcome i of the preparation process, just that it was, by design, the same outcome in each shot. If we want to retrodict the prepared state of a quantum system we should write the state in terms of those states which could possibly have been prepared, so in general we write this density operator after N shots in the form


(4.3) $${\hat{\rho }}^{\left(N\right)}=\sum_{i}p_{i}^{\left(N\right)}{\hat{\rho }}_{i}^{\text{ pred }},$$

where $p_{i}^{\left(N\right)}$ is a representation of our belief that the state ${\hat{\rho }}_{i}^{\text{ pred }}$ was prepared after N shots of the experiment. Equation (2.2) is one example of this formula, after zero shots of the experiment have been performed so that $p_{i}^{\left(0\right)}=p_{i}^{\text{ ap }}$. This formulation provides a preferred ensemble for retrodiction of the preparation event [50].^2^ We now proceed to show how the $p_{i}^{\left(N\right)}$ are calculated after N shots of the experiment.

We calculate the predictive conditional probabilities of particular measurement results j if the preparation event was i via equation (2.4). Similarly, the overall probability that we get the measurement result j without knowing the preparation outcome i is


(4.4) $$p(j)=\sum_{i}p_{i}^{\left(0\right)}Tr({\hat{\rho }}_{i}^{\text{ pred }}{\hat{\pi }}_{j})=Tr({\hat{\rho }}^{\left(0\right)}{\hat{\pi }}_{j}),$$

with ${\hat{\rho }}^{\left(0\right)}$ the initial *a priori* density operator from equation (2.2), with the superscript (0) to tell us that it has not been updated. Bayes’ theorem provides the retrodictive conditional probability $p^{\left(1\right)}(i|j)$, the probability that the prepared state was ${\hat{\rho }}_{i}$ given that the first measurement outcome was j,


(4.5) $$\begin{array}{ccc} & p^{\left(1\right)}(i|j)=\frac{p_{i}^{\left(0\right)}p(j|i)}{\sum_{k}p_{k}^{\left(0\right)}p(j|k)}=\frac{p_{i}^{\left(0\right)}\mathrm{Tr}({\hat{\rho }}_{i}{\hat{\pi }}_{j})}{\sum_{k}p_{k}^{\left(0\right)}\mathrm{Tr}({\hat{\rho }}_{k}{\hat{\pi }}_{j})}=\frac{p_{i}^{\left(0\right)}\mathrm{Tr}({\hat{\rho }}_{i}{\hat{\pi }}_{j})}{\mathrm{Tr}({\hat{\rho }}^{\left(0\right)}{\hat{\pi }}_{j})}. & \end{array}$$

This set of conditional probabilities is of significant interest to us in that it can be inserted into equation (4.3) to provide a new density operator that includes not only the prior information, but also the update based on our measurement result, ${\hat{\pi }}_{j}$, via


(4.6) $$\begin{array}{ccc} & {\hat{\rho }}_{j}^{\left(1\right)}=\sum_{i}p^{\left(1\right)}(i|j){\hat{\rho }}_{i}. & \end{array}$$

This is now the best guess of the prepared state that we can make without knowledge of the preparation outcome. It is an update on the *a priori* density operator, which formed this best guess prior to a shot of the experiment. It forms a kind of retrodicted prepared state, an updated version after a measurement. In general it is not the same as the retrodictive state, which is based solely on the single-shot measurement result.

### (c) Repeat experiments with prior information

We have seen in the previous section that Bayes’ theorem allows us to define a new best guess state after one experimental shot even if we have prior information about the states that could have been prepared. At the first measurement outcome $j_{1}$ occurs, which changes our knowledge of the prepared state to that given by equation (4.6) with $j_{1}$ substituted for j.

We now have a choice—almost a philosophical one. We could treat the second shot of the experiment, with outcome $j_{2}$, as independent of the first, with the same prior. If the outcome $j_{2}$ is different from $j_{1}$ the best guess for the state will be of similar form to equation (4.6), but will represent a different state. We could sum the two best guess states and normalize, in a similar manner to the procedure that produced equation (4.2). We then continue in the same fashion for any later shots of the experiment. Such a procedure will not always converge on to one of the prepared states (e.g. if the possible prepared states are not all orthogonal) because it introduces frequentism into our previous Bayesian reasoning.

Instead we take Bayes’ theorem seriously and use the updated state from the first measurement, equation (4.6), as our *a priori* state for the second shot of the experiment. All of the predictive conditional probabilities remain the same, but the retrodictive ones become


(4.7) $$\begin{array}{ccc} & p^{\left(2\right)}(i|j_{1},j_{2})=\frac{p_{i}^{\left(1\right)}\mathrm{Tr}({\hat{\rho }}_{i}{\hat{\pi }}_{j_{2}})}{\sum_{k}p_{k}^{\left(1\right)}\mathrm{Tr}({\hat{\rho }}_{k}{\hat{\pi }}_{j_{2}})}=\frac{p_{i}^{\left(1\right)}\mathrm{Tr}({\hat{\rho }}_{i}{\hat{\pi }}_{j_{2}})}{\mathrm{Tr}({\hat{\rho }}_{j_{1}}^{\left(1\right)}{\hat{\pi }}_{j_{2}})}, & \end{array}$$

where $p_{i}^{\left(1\right)}=p^{\left(1\right)}(i|j_{i})$ is the ‘prior’ probability after one measurement result. We can use the probabilities $p^{\left(2\right)}(i|j_{1},j_{2})$ to define a new best guess state for the preparation after two shots of the experiment,


(4.8) $$\begin{array}{ccc} & {\hat{\rho }}_{j_{1},j_{2}}^{\left(2\right)}=\sum_{i}p^{\left(2\right)}(i|j_{1},j_{2}){\hat{\rho }}_{i}. & \end{array}$$

The extension to multiple measurements is straightforward. After N measurements the state is


(4.9) $$\begin{array}{ccc} & {\hat{\rho }}_{j_{1},...j_{N}}^{\left(N\right)}=\sum_{i}p^{\left(N\right)}(i|j_{1},...j_{N}){\hat{\rho }}_{i}. & \end{array}$$

By this method, we obtain a gradually improving guess of the prepared state that will eventually arrive at the state that was repeatedly prepared.

#### (i) Example: estimation of photonic states

Our example is based on imperfect measurements of optical fields with photodetectors. In some cases, our formulation allows imperfect measurements to make a perfect retrodiction of the prepared state when a perfect detector does not. Fundamentally this is a problem of estimating states of the electromagnetic field using informationally incomplete measurements. Consider a single-mode source that can be prepared in one of three Fock states, {|0⟩,|1⟩,|2⟩}. We assume that each state is prepared with equal probability. The single mode is measured using a threshold detector with efficiency η. This measurement has two outcomes: no photons detected (no-click) and photons detected (click). The probability operator for no-click is ${\hat{\pi }}_{0}=\sum_{n=0}^{2}(1-\eta {)}^{n}|n\rangle \langle n|$, while the click probability operator is ${\hat{\pi }}_{1}=\hat{1}-{\hat{\pi }}_{0}$. These lead to retrodictive states given by the state-space limited and renormalized equation (3.6) and its similarly limited renormalized complement


(4.10) $$\begin{array}{lll} {\hat{\rho }}_{0}^{\text{ retr }} & = & \frac{1}{3-3\eta +\eta^{2}}\sum_{n=0}^{2}(1-\eta {)}^{n}|n\rangle \langle n|,\\ {\hat{\rho }}_{1}^{\text{ retr }} & = & \frac{|1\rangle \langle 1|+(2-\eta )|2\rangle \langle 2|}{3-\eta }, \end{array}$$

corresponding to the amplified vacuum and complement vacuum states. We use these measurements to condition the *a priori* density operator repeatedly via equation (4.9).

**Figure 4.**
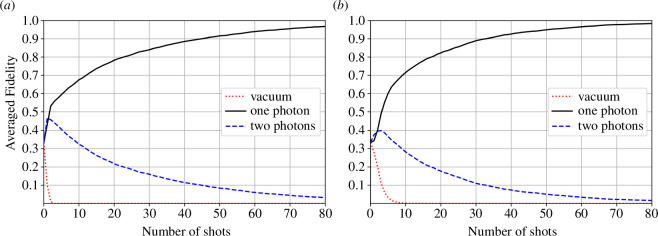
Averaged fidelity (3000 runs) between the retrodictive estimate and the states |0⟩ (dotted red line), |1⟩ (solid black line) and |2⟩ (dashed blue line), against the number of measured copies with (*a*) η=0.9 and (*b*) η=0.5.

We perform a Monte Carlo simulation in which |1⟩ is repeatedly prepared, averaged over 3000 runs. Figure 4 shows the averaged fidelity of the best guess of the prepared state, from equation (4.9), against number of detected states N. The fidelities are calculated for each of the three possible prepared states {|0⟩,|1⟩,|2⟩}. In the two panels, the quantum efficiencies are (i) η=0.9 and (ii) η=0.5. Both (i) and (ii) show similar trends in that the one-photon fidelity increases with the number of copies, which demonstrates that measurement is able to estimate the state accurately. The zero-photon fidelity rapidly decays and this occurs faster for η=0.9. This is because a single click is enough to indicate that the state is not the vacuum. An interesting feature is that for small values of N, both the one- and two-photon fidelities increase, until eventually the two-photon fidelity begins to decay. In fact, initially the two-photon fidelity increases faster than the one-photon fidelity. To understand this behaviour, we can examine the retrodictive states, equation (4.10). If we register a click for low N, $\left\langle 2|{\hat{\rho }}_{1}^{\text{ retr }}|2\right\rangle$ is greater than $\left\langle 1|{\hat{\rho }}_{1}^{\text{ retr }}|1\right\rangle$, which means a click is more likely to be associated with the state |2⟩ when η<1. However, after we are confident the state was not the vacuum, then not registering a click makes us more confident that the state is actually the single-photon state, rather than a two-photon state.

Another strange feature of figures 4*a,b* is that for large N, the fidelity of the correct state |1⟩ is greater for the poorer detector, η=0.5, than for η=0.9. A poorer detector seems to identify the one-photon state more quickly. The same would be true if we had prepared the two-photon state—a poorer detector would identify it more quickly. In fact there is an optimal value of η between 0 and 1 that can be found from the retrodictive states that provides the best distinguishability between the one- and two-photon states. Equation (4.10) shows that as η→0, i.e. the feeble detector does not fire, no information is gained. On the other hand, as η→1, a single firing excludes the vacuum state, but firing does not distinguish between the one- and two-photon states. So the efficiency η must be large enough for the detector to fire occasionally if photons are present. When it does not fire it preferentially picks out the one-photon state (the weighting of the two-photon state is 1−η smaller than that of the one-photon state in ${\hat{\rho }}_{0}^{\text{ retr }}$). The efficiency must also be sufficiently smaller than 1 so that it can preferentially pick out the higher weighted two-photon state in ${\hat{\rho }}_{1}^{\text{ retr }}$ when the detector fires. This is the trade-off that determines the best value of efficiency. The interplay between these two efficiency criteria suggests that an adaptive Bayesian strategy may be better in principle [51], for example with a high value of η used until the first click and then a lower one afterwards.

### (d) Independent state choice

Finally we examine the case of choosing an independent state each time we do the experiment. A single shot of the experiment provides the same results as §4a. The best guess of the prepared state is given by equation (4.6), with the conditional probabilities given by equation (4.5). The differences begin when we consider the second shot of the experiment. Because the preparation outcome is in principle different (say $i_{2}$) the second experiment does not necessarily tell us anything about the first preparation. We cannot use either to update the prior probabilities in the *a priori* density operator. After many shots of the experiment the detection results will correspond simply to a detection-probability-weighted sum of the retrodictive states. The weightings will be such that the sum corresponds to the *a priori* density operator, but written in the retrodictive basis, not the possible prepared states of the preparation basis.

## 5. Conclusions

Retrodictive quantum mechanics is almost 70 years old at the time of writing. In its modern form, couched in the language of modern quantum measurement theory, it has been a useful tool for the development of quantum optics and information. Even the spur for its resurgence, the theoretical proposal of the quantum scissors device in 1998, led not only to a physical realization, but also an unforeseen development of new forms of postselecting quantum amplifiers that beat the traditional noise addition limit [52].

The first formulations of retrodiction were based on ensemble averages and so were more akin to frequentism [4]. As quantum physics began to be applied to single systems this was no longer adequate. If the state of a single quantum system can be thought of as a subjective entity that depends on the information that an outside observer (preparer or measurer) has, then many states can be defined that are consistent with the probabilistic mathematics of quantum theory. Bayes’ theorem provides a natural relation between two sets of these states, the predictive ones produced by a preparation device and the retrodictive states that are measured, as it does for classical probabilities [10].

In this paper, we have reviewed the main results of retrodictive quantum mechanics, before extending the theory towards an ensemble-based past, via independent measurements on multiple versions of the same prepared state. The formalism allows an observer to retrodict the prepared state from a knowledge of the states that could have been prepared and a set of measurement results that do not distinguish them perfectly (or even very well, as our photon example shows). This retrodicted prepared state forms a natural multi-shot-based counterpart to the *a priori* state. We will apply this theory further, for example, to measuring non-orthogonal states on the Bloch sphere, elsewhere.

## Data Availability

This article has no additional data.

## Footnotes

^1^
The set of generalized measurement operators goes by several names, of which two are probability operator measure and positive operator-valued measure. We prefer here the term probability operators as it is descriptive of what they provide.

^2^
Conversely, if we want to predict the results of measurements or, more specifically, the state corresponding to the possible measurement results it is calculationally advantageous to write the state in terms of those measurement results.

## References

[1] Bayes T. 1763 An essay towards solving a problem in the doctrine of chances. Phil. Trans. Roy. Soc. **53**, 370–418. (10.1098/rstl.1763.0053) 1857193

[2] Laplace PS. 1774 Mémoire sur la probabilité des causes par les événements. Mém. Acad. Roy. Sci. MI **4**, 621–656. https://www.jstor.org/stable/2245475

[3] McGrane SB. 2011 The theory that would not die. New Haven, CT and London, UK: Yale University Press.

[4] Watanabe S. 1955 Symmetry of physical laws. Part III. Prediction and retrodiction. Rev. Mod. Phys. **27**, 179–186. (10.1103/RevModPhys.27.179)

[5] Aharonov Y, Bergmann PG, Lebowitz JL. 1964 Time symmetry in the quantum process of measurement. Phys. Rev. **134**, B1410–B1416. (10.1103/PhysRev.134.B1410)

[6] Penfield RH. 1966 More on the arrow of time. Am. J. Phys. **34**, 422–426. (10.1119/1.1973012)

[7] Pegg DT, Phillips LS, Barnett SM. 1998 Optical state truncation by projection synthesis. Phys. Rev. Lett. **81**, 1604–1606. (10.1103/PhysRevLett.81.1604)

[8] Barnett SM, Phillips LS, Pegg DT. 1998 Imperfect photodetection as projection onto mixed states. Opt. Commun. **158**, 45–49. (10.1016/S0030-4018(98)00511-2)

[9] Pegg DT, Barnett SM. 1999 Retrodiction in quantum optics. J. Opt. B Quantum Semiclass. Opt. **1**, 442–445. (10.1088/1464-4266/1/4/314)

[10] Barnett SM, Pegg DT, Jeffers J. 2000 Bayes’ theorem and quantum retrodiction. J. Mod. Opt. **47**, 1779–1789. (10.1080/09500340008232431)

[11] Barnett SM, Pegg DT, Jeffers J, Jedrkiewicz O. 2000 Atomic retrodiction. J. Phys. B At. Mol. Opt. Phys. **33**, 3047–3065. (10.1088/0953-4075/33/16/309)

[12] Jeffers J, Barnett SM, Pegg DT. 2002 Retrodiction with two-level atoms: atomic previvals. J. Mod. Opt. **49**, 1175–1184. (10.1080/09500340110100592)

[13] Barnett SM, Pegg DT, Jeffers J, Jedrkiewicz O, Loudon R. 2000 Retrodiction for quantum optical communications. Phys. Rev. A **62**, 022213. (10.1103/PhysRevA.62.022313) 11289953

[14] Jedrkiewicz O, Jeffers J, Loudon R. 2000 Retrodiction for optical attenuators, amplifiers, and detectors. Phys. Rev. A **70**, 033805. (10.1103/PhysRevA.70.033805)

[15] Jedrkiewicz O, Loudon R, Jeffers J. 2006 Retrodiction for coherent communication with homodyne or heterodyne detection. Eur. Phys. J. D **39**, 129–140. (10.1140/epjd/e2006-00081-7)

[16] Jeffers J, Barnett SM, Pegg DT. 2002 Retrodiction as a tool for micromaser field measurements. J. Mod. Opt. **49**, 925–938. (10.1080/09500340110110069)

[17] Tan EK, Jeffers J, Barnett SM. 2004 Field-state measurement in a micromaser using retrodictive quantum theory. Phys. Rev. A **69**, 043806. (10.1103/PhysRevA.69.043806)

[18] Pregnell KL, Pegg DT. 2004 Retrodictive quantum optical state engineering. J. Mod. Opt. **51**, 1613–1626. (10.1080/09500340408232476)

[19] Pregnell KL. 2005 Retrodictive quantum state engineering. (10.48550/arXiv.quant-ph/0508088)

[20] Jeffers J. 2006 Retrodictive fidelities for pure state postselectors. New J. Phys. **8**, 268–268. (10.1088/1367-2630/8/11/268)

[21] Chefles A, Sasaki M. 2003 Retrodiction of generalized measurement outcomes. Phys. Rev. A **67**, 032112. (10.1103/PhysRevA.67.032112)

[22] Tan EK, Jeffers J, Barnett SM, Pegg DT. 2003 Retrodictive states and two-photon quantum imaging. Eur. Phys. J. D **22**, 495–499. (10.1140/epjd/e2003-00006-0)

[23] Sonnleitner M, Jeffers J, Barnett SM. 2015 Image retrodiction at low light levels. Optica **2**, 950. (10.1364/OPTICA.2.000950)

[24] Sonnleitner M, Jeffers J, Barnett SM. 2016 Local retrodiction models for photon-noise-limited images. In SPIE Photonics Europe, Brussels, Belgium, vol. 9896, p. 203, (10.1117/12.2224444)

[25] Speirits FC, Sonnleitner M, Barnett SM. 2017 From retrodiction to Bayesian quantum imaging. J. Opt. **19**, 044001. (10.1088/2040-8986/aa5ccf)

[26] Johnstone GE, Herrnsdorf J, Dawson MD, Strain MJ. 2023 Efficient reconstruction of low photon count images from a high speed camera. Photonics **10**, 10. (10.3390/photonics10010010)

[27] Kalev A, Mann A, Revzen M. 2013 Quantum-mechanical retrodiction through an extended mean king problem. EPL **104**, 50008. (10.1209/0295-5075/104/50008)

[28] Barnett SM, Cresser JD, Jeffers J, Pegg DT. 2014 Quantum probability rule: a generalization of the theorems of Gleason and Busch. New J. Phys. **16**, 043025. (10.1088/1367-2630/16/4/043025)

[29] Yuan Y, Hou Z, Wu KD, Xiang GY, Li CF, Guo GC. 2018 Experimental retrodiction of trajectories of single photons in double interferometers. Phys. Rev. A **97**, 062115. (10.1103/PhysRevA.97.062115)

[30] Pegg DT, Barnett SM, Jeffers J. 2002 Quantum theory of preparation and measurement. J. Mod. Opt. **49**, 913–924. (10.1080/09500340110109412)

[31] Pegg DT, Barnett SM, Jeffers J. 2002 Quantum retrodiction in open systems. Phys. Rev. A **66**, 022106. (10.1103/PhysRevA.66.022106)

[32] Barnett SM, Pegg DT, Jeffers J, Jedrkiewicz O. 2001 Master equation for retrodiction of quantum communication signals. Phys. Rev. Lett. **86**, 2455–2458. (10.1103/PhysRevLett.86.2455) 11289953

[33] Barnett SM, Jeffers J, Pegg DT. 2021 Quantum retrodiction: foundations and controversies. Symmetry **13**, 586. (10.3390/sym13040586)

[34] Aw CC, Buscemi F, Scarani V. 2021 Fluctuation theorems with retrodiction rather than reverse processes. AVS Quantum Sci. **3**, 045601. (10.1116/5.0060893)

[35] Buscemi F, Schindler J, Šafránek D. 2023 Observational entropy, coarse-grained states, and the Petz recovery map: information-theoretic properties and bounds. New J. Phys. **25**, 053002. (10.1088/1367-2630/accd11)

[36] Tsang M. 2022 Generalized conditional expectations for quantum retrodiction and smoothing. Phys. Rev. A **105**, 042213. (10.1103/PhysRevA.105.042213)

[37] Carette J, Ortiz G, Sabry A. 2022 Retrodictive quantum computing. (10.48550/arXiv.2205.06346)

[38] Parzygnat AJ, Buscemi F. 2023 Axioms for retrodiction: achieving time-reversal symmetry with a prior. Quantum **7**, 1013. (10.22331/q-2023-05-23-1013)

[39] Fitzke E, Niederschuh F, Walther T. 2023 Simulating the photon statistics of multimode Gaussian states by automatic differentiation of generating functions. APL. Photonics **8**, 026106. (10.1063/5.0129638)

[40] Box GEP, Tiao GC. 1973 Bayesian inference in statistical analysis. Sydney, Australia: Addison-Wesley.

[41] Ban M. 2007 Quantum retrodiction in non-equilibrium thermo field dynamics. Int. J. Theor. Phys. **46**, 184–192. (10.1007/s10773-006-9228-7)

[42] Scroggie AJ, Jeffers J. 2008 A closed-system approach to quantum retrodiction in open systems. Int. J. Theor. Phys. **47**, 1809–1816. (10.1007/s10773-007-9623-8)

[43] Haus HA, Mullen JA. 1962 Quantum noise in linear amplifiers. Phys. Rev. **128**, 2407–2413. (10.1103/PhysRev.128.2407)

[44] Caves CM. 1982 Quantum limits on noise in linear amplifiers. Phys. Rev. D **26**, 1817–1839. (10.1103/PhysRevD.26.1817)

[45] Loudon R. 1973 The quantum theory of light, 1st edn. Oxford, UK: Oxford University Press.

[46] Loudon R. 1983 The quantum theory of light, 2nd edn. Oxford, UK: Oxford University Press.

[47] Glauber RJ. 1986 Frontiers in quantum optics. Bristol, UK: Adam Hilger.

[48] Loudon R. 2000 The quantum theory of light, 3rd edn. Oxford, UK: Oxford University Press.

[49] Jeffers J, Imoto N, Loudon R. 1993 Quantum optics of traveling-wave attenuators and amplifiers. Phys. Rev. A **47**, 3346–3359. (10.1103/physreva.47.3346) 9909314

[50] Pegg DT, Jeffers J. 2005 Quantum nature of laser light. J. Mod. Opt. **52**, 1835. (10.1080/09500340500106857)

[51] Oi DKL, Schirmer SG. 2012 Quantum system characterization with limited resources. Phil. Trans. R. Soc. A **370**, 5386–5395. (10.1098/rsta.2011.0530) 23091215

[52] Ralph TC, Lund AP. 2009 Nondeterministic noiseless linear amplification of quantum systems (ed. A Lvovsky). In Quantum Communication Measurement and Computing Proceedings of 9th International Conference, p. 155. New York: AIP.

